# Psychological determinants of participation in the sharing economy: a cross-cultural study of the U.S. and China

**DOI:** 10.3389/fpsyg.2025.1666282

**Published:** 2026-01-16

**Authors:** Jia Song, Sami Kajalo

**Affiliations:** 1 School of Management, Shenzhen Polytechnic University, Shenzhen, China; 2 Centre for Modern Industry and SME, Shenzhen, China; 3 Aalto University School of Business, Espoo, Finland

**Keywords:** consumer psychology, sharing economy, structural equation modeling, theory of reasoned action, value–attitude–behavior

## Abstract

**Introduction:**

This study explores the psychological drivers of participation in the sharing economy through a cross-cultural lens, comparing consumer behavior in the United States and China.

**Methods:**

Drawing on the value–attitude–behavior hierarchy theory and the theory of reasoned action, we investigate how utilitarian, hedonic, and symbolic values shape attitudes, norms, and behavioral intentions in the context of collaborative consumption. Data were collected through online surveys from 302 participants in the U.S. and 305 in China and analyzed using Structural Equation Modelling (SEM).

**Results:**

Our findings reveal both similarities and differences in how personal values influence consumer decision-making. While utilitarian and hedonic values positively affect attitudes in both countries, symbolic value does not. Norms play culturally distinct roles: in the U.S., subjective norms are more predictive of intention, whereas in China, personal norms exert a stronger influence.

**Discussion:**

These results underscore the need for culturally adaptive strategies in the design and marketing of sharing economy platforms. The study contributes to cross-cultural consumer psychology by clarifying how values and norms shape collaborative consumption across distinct cultural contexts.

## 1 Introduction

The sharing economy is reshaping how people consume. Individuals can share goods, intangible assets, and unused resources (Kathan et al., 2016) by offering temporary access to other consumers (Eckhardt et al., 2019). As a concept, the sharing economy lacks clear boundaries (Hossain, 2020). It has also been referred to as shared consumption (Roos and Hahn, 2017), collaborative consumption (Barnes and Mattsson, 2017), collaborative economy (Martin, 2016), collective consumption (Hamari et al., 2016), access-based consumption (Bardhi and Eckhardt, 2012), and other related terms, all of which have received significant attention in recent years. In the sharing economy process, individuals act as both consumers and providers of services (Nakamura et al., 2021). The growing sharing economy now affects consumers’ lives in many ways through its flexible business models.

Research on consumers of the sharing economy spans various fields, including accommodation sharing (Lutz and Newlands, 2018), bike-sharing (Ma et al., 2018), ride-sharing (Cheng et al., 2018), and luxury goods sharing (Pantano and Stylos, 2020), among others. The Sharing Economy Association Japan defines the sharing economy in five categories, namely, skills, goods, space, mobility, and money (Yamasawa, 2018). In China, the Research Center for the Sharing Economy at the National Information Center publishes annual reports that analyze ride-sharing, accommodation-sharing, knowledge and skills exchange, shared life services, shared healthcare, co-working spaces, and production capacity (State Information Center, 2023). These domains encompass various aspects of daily life and enable many companies to offer new and varied services. For instance, entire homes, private rooms, and shared rooms are available on Airbnb (Lutz and Newlands, 2018). In this way, sharing economy firms have expanded internationally at incredible speed (Parente et al., 2018).

Growing research has explored the sharing economy from various perspectives, including concept construction (Acquier et al., 2017), characteristics of the sharing economy (Petruzzi et al., 2021), digital platforms (de Rivera et al., 2017), consumer response to pricing (Dowling et al., 2020), and more. Moreover, the concept of the sharing economy has also been debated in different societal contexts. For instance, Japanese researchers view it as a tool that could help mitigate the depopulation of rural areas and address labor shortages in an aging society (Majima et al., 2021). However, there is a dearth of studies examining the role of personal factors in the sharing economy (Gupta and Chauhan, 2021), and what motivates individuals to participate remains unclear (Huang and Kuo, 2020). Moreover, several studies have already tested the effect of different types of values. For example, biospheric and egoistic values have been confirmed to have no statistically significant effects on the sharing economy, while altruistic value has a statistically significant positive impact (Roos and Hahn, 2017). In addition, the specific dimensions of perceived value (utilitarian, hedonic, and symbolic) have differing impacts on young consumers’ attitudes and empathy toward the sharing economy (Hwang and Griffiths, 2017). Whether these value dimensions affect norms and attitudes in different cultural contexts is still not well understood. Research at this level remains underexplored.

To address these research gaps, this study focuses on the following research questions:

1. What factors influence individuals’ willingness to participate in the sharing economy?
2. What is the relationship between values, norms, and attitudes that affect customer intention in the sharing economy?
3. How do these relationships differ between the U.S. and China?
4. How can these insights guide platform strategies in different markets?

To answer these questions, this study employs a quantitative approach to investigate the factors that influence consumers’ intentions within the sharing economy. Specifically, this study examines the sharing economy within the contexts of the U.S. and China. Drawing on the value–attitude–behavior (VAB) hierarchy theory (Homer and Kahle, 1988), the theory of reasoned action (TRA) (Ajzen and Fishbein, 1980), and the theory of planned behavior (Ajzen, 1991), this paper adopts a consumer-centric perspective and sheds light on the relationships among individual-level factors.

Thus, this study offers multiple contributions to both theory and practical applications. Firstly, it adds to the existing literature on the sharing economy. Secondly, the study not only confirms but also expands upon previous research regarding consumer psychology. Additionally, it offers practical insights by analyzing how utilitarian, hedonic, and symbolic values influence consumers’ norms and attitudes. It helps sharing economy marketers better align with consumers’ personal expectations.

The structure of the paper is as follows: we start by examining the value–attitude–behavior hierarchy theory along with the theory of reasoned action, specifically within the framework of the sharing economy. We then construct the hypotheses via value–attitude–intention and value–norm–intention paths. Next, we describe the data collection process and analytical method. We then present our findings, followed by discussion and implications. Finally, the conclusion and limitations of the contribution will be discussed.

## 2 Theory background and hypotheses development

The sharing economy has brought notable benefits to consumers and society. It helps reduce unethical behavior (Guo et al., 2019), lowers transaction costs (Huarng, 2018), decreases carbon emissions and other ecological impacts (Kim et al., 2018), minimizes pollutants (Dabbous and Tarhini, 2021), creates new income opportunities (Räisänen et al., 2021), and enhances individual quality of life (Huynh et al., 2020). However, several studies also highlight negative consequences. These include clashes with local competition and regulators (Parente et al., 2018) as well as economic, social, and psychological costs for participants and third parties (Köbis et al., 2021). Increasingly, previous studies emphasize the sharing economy’s role in promoting sustainability and eco-conscious consumption (Zhu and Liu, 2021; Curtis and Mont, 2020).

Various factors influencing sharing economy participation have attracted scholarly attention. Key themes include trust (Celata et al., 2017), social presence (Cho et al., 2019), national cultural values (Gupta et al., 2019), reputation (Mikołajewska-Zając, 2018), sustainability, enjoyment, and economic gains (Hamari et al., 2016), extrinsic and intrinsic motivation (Li and Wen, 2019), financial drivers (Billows and McNeill, 2018), and social connection (Boateng et al., 2019). Other studies have explored customer loyalty in sharing services (Yang et al., 2017) and inhibitors such as resistance and anxiety related to sharing with strangers (Nakamura et al., 2021). While many factors have been studied, the role of values remains relatively underexplored. In particular, the value dimensions that support participation in the sharing economy warrant further investigation.

### 2.1 Value and attitude

The value–attitude–behavior (VAB) hierarchy theory, introduced by Homer and Kahle (1988), offers a structured framework for understanding how personal values influence attitudes and ultimately behavior. Values are defined as trans-situational, enduring beliefs about desirable end states or modes of conduct (Rokeach, 1973). These constructs guide perception and decision-making over time, shaping attitudes and behavioral tendencies (Bergman, 1998).

In the context of the sharing economy, user motivations have been linked to utilitarian, hedonic, and symbolic values (Hepola et al., 2020; Hwang and Griffiths, 2017; Lee and Kim, 2018; Miao et al., 2014). Utilitarian values are rooted in function and efficiency: consumers guided by these values often lead simpler lifestyles, and seek practical benefits from consumption (Wang and Lin, 2009). In the sharing economy, such practical advantages include cost savings (Hamari et al., 2016). For example, convenience and economic efficiency are dominant in car-sharing platforms (Bardhi and Eckhardt, 2012). Airbnb offers hosts a source of income and provides customers with more affordable and unique experiences compared to hotels (Lee and Kim, 2018; Guttentag, 2015).

Hedonic value, by contrast, involves emotional and experiential rewards. It reflects a consumer’s desire for novelty, pleasure, and enjoyment (Tsou et al., 2019). Staying in unique accommodations such as igloos, castles, or treehouses adds experiential richness (Miao et al., 2014), while renting luxury attire for special events enhances emotional value (Pantano and Stylos, 2020).

Symbolic value refers to non-material meanings embedded in consumption, often tied to self-image, identity, and social affiliation (Hasan, 2022). Consumers may choose sharing services to express pro-environmental values or project socially responsible identities. For example, by ride-sharing to reflect sustainability concerns (Gadeikiene and Svarcaite, 2021).

The theory of reasoned action (TRA) (Ajzen and Fishbein, 1980) and the theory of planned behavior (TPB) (Ajzen, 1991) explains attitude as a person’s evaluation of a behavior as positive or negative. Attitudes are shaped by beliefs and previous experiences and play a central role in predicting intentions (Ajzen and Fishbein, 1980; Lo et al., 2020). Attitudes have been identified as one of the strongest predictors of sharing economy participation (Akande et al., 2020), and their influence may vary depending on prior experience (Tanveer et al., 2025).

Building on the main ideas of these three theories (VAB, TRA, and TPB), and on existing research in the sharing economy, this study follows the logic of the value–attitude chain. Value is regarded as an antecedent of attitude in various fields (Chang et al., 2020). We propose that utilitarian, hedonic, and symbolic values are the main factors influencing the user’s attitude. Therefore, we expect these three core values to shape attitudes toward the benefits of the sharing economy, which can encourage increased participation.

Thus we propose:

H1a: Utilitarian value positively affects attitude toward the benefits of the sharing economy.

H1b: Hedonic value positively affects attitude toward the benefits of the sharing economy.

H1c: Symbolic value positively affects attitude toward the benefits of the sharing economy.

### 2.2 Subjective norm and personal norm

Subjective norm refers to an individual’s perception of social pressure to perform or avoid a particular behavior (Ajzen, 1991). These norms stem from normative beliefs about how significant others (e.g., friends, family, or peers) expect them to behave, and the extent to which individuals are motivated to comply (Park, 2000; Moser, 2016).

In the context of the sharing economy, particularly Airbnb, three subjective norms have been identified as influential: peer influence, external influence, and word-of-mouth (Tajeddini et al., 2021). Peer influence is rooted in the behavior and opinions of one’s social group (Schiffman and Kanuk, 2007). For instance, individuals may follow friends’ recommendations when booking shared accommodation (Barnes and Mattsson, 2017). External influence refers to credible input from strangers (e.g., expert reviews or media) that shapes perceptions (Cohen et al., 2014). Word-of-mouth often comes through online reviews, where users evaluate others’ feedback to guide their own decisions (Xu, 2020; Lee and Wong, 2021). These social cues help shape behavioral expectations (Małecka et al., 2022).

In contrast, personal norms are internally held moral obligations (Thøgersen, 2009; de Groot et al., 2021). When individuals view certain behaviors as consistent with their ethical standards or identity, personal norms strongly predict intention. For example, those with high moral identity may reduce car use or prefer sustainable alternatives like bike-sharing (Ricci, 2015; Culiberg et al., 2023). Rental users (e.g., Uber or Airbnb users) often express pro-sustainable identities rooted in personal values (Shukla et al., 2024). While subjective norm represents external social pressure, personal norm reflect internal moral duty (Roos and Hahn, 2019). Research shows that the more robust a person’s personal norm related to pro-environmental behavior, the stronger their corresponding intentions and actions tend to be (Joanes, 2019; Onwezen et al., 2013).

**Figure 1:**
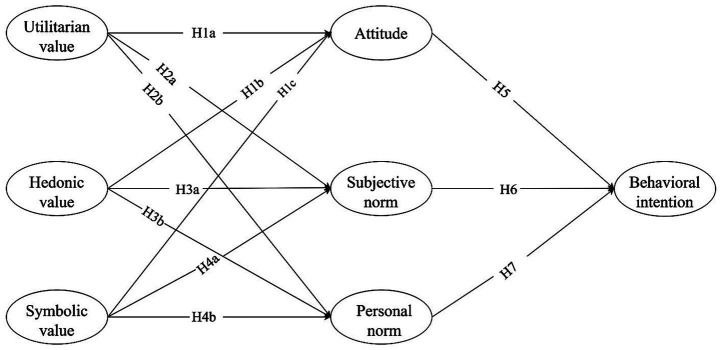
The conceptual framework of the study.

In our conceptual framework, following the prior research, subjective norm is the key variable for customer participation (Blut and Wang, 2025), and personal norms that are separate from social norms across various economic contexts (Bašić and Verrina, 2024). Perceived moral obligation may influence the subjective norm and personal norm differently (Fauzi et al., 2024). Regarding the sharing economy, the values can also indirectly encourage behavior by shaping subjective and personal norms (see Figure 1).

**Figure 2:**
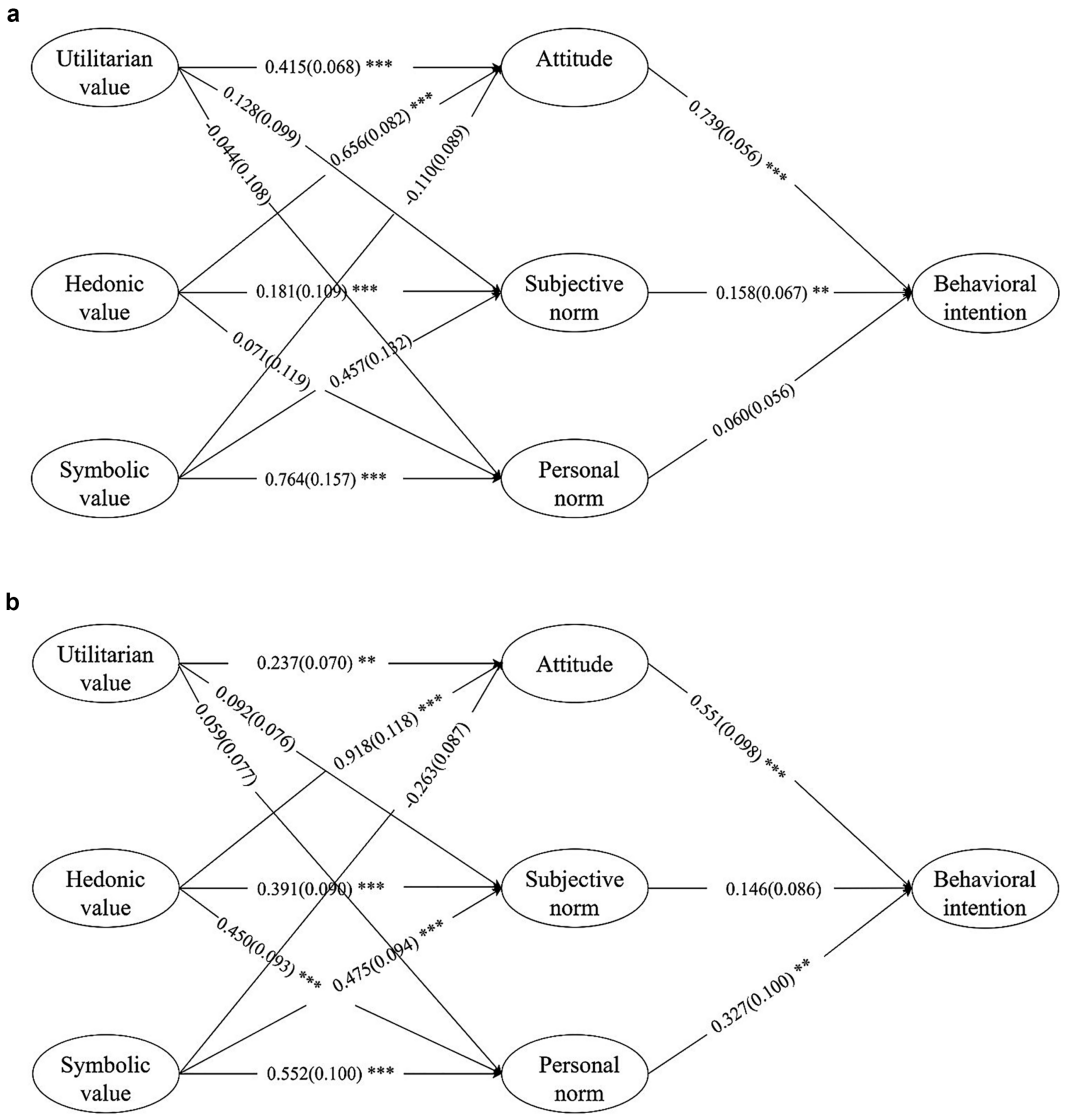
**(a)** The final model of the U.S. data: results of the final model with standardized (SE in parentheses) estimates. *n* = 302. *p* < 0.001, 90% CI (0.079, 0.093), SRMR = 0.0596. Correlations between exogenous variables are not shown. **p < 0.01; ***p < 0.001. **(b)** The final model of the China data: results of the final model with standardized (SE in parentheses) estimates. *n* = 305. *p* < 0.001, 90% CI (0.065, 0.079), SRMR = 0.0735. Correlations between exogenous variables are not shown. ^**^p < 0.01; ^***^p < 0.001.

Thus, we hypothesize:

H2a: Utilitarian value positively affects subjective norm toward the benefits of the sharing economy.

H2b: Utilitarian value positively affects personal norm toward the benefits of the sharing economy.

H3a: Hedonic value positively affects subjective norm toward the benefits of the sharing economy.

H3b: Hedonic value positively affects personal norm toward the benefits of the sharing economy.

H4a: Symbolic value positively affects subjective norm toward the benefits of the sharing economy.

H4b: Symbolic value positively affects personal norm toward the benefits of the sharing economy.

### 2.3 Norms, attitude, and intention

Both subjective and personal norms influence behavior not only directly but also through their impact on attitudes (Smetana and Adler, 1980). According to the theory of reasoned action, intentions are shaped by attitudes and subjective norm (Ajzen and Fishbein, 1980). Multiple studies confirm that attitude is a key predictor of behavioral intention in the sharing economy (Akande et al., 2020; Chen and Ryu, 2024).

Empirical work further shows that enhancing consumer perceptions and experiences improves reuse intention (Elnadi and Gheith, 2022), and both attitude and social norms influence consumer decisions in services like fashion rental (Lee and Chow, 2020). Additionally, studies in transportation-sharing contexts have demonstrated a strong positive impact of attitude on behavioral intention (Huang et al., 2021; Jing et al., 2021). Carsharing users often view it as an eco-friendly transportation option because it helps reduce many negative impacts of automobility (Vejchodská et al., 2024), and provides a sense of belonging (Schaefers, 2013).

In peer-to-peer accommodations, moral obligations (i.e., personal norms) also influence intention alongside attitudes and subjective norms (Shin et al., 2023). Previous studies have shown that personal norm positively influences intention (Tandon et al., 2023; Wang et al., 2023). In this study, we integrate subjective norm, personal norm, and attitude into a unified research framework. Attitude refers to the consumer’s overall evaluation of sharing economy participation, including its social, environmental, and economic benefits. In line with circular economy principles, these attitudes are expected to play a central role in predicting intention.

We therefore hypothesize:

H5: Attitude positively affects behavioral intention toward the benefits of the sharing economy.

H6: Subjective norm positively affects behavioral intention toward the benefits of the sharing economy.

H7: Personal norm positively affects behavioral intention toward the benefits of the sharing economy.

## 3 Research method

### 3.1 Data collection

This study explores engagement in the sharing economy to provide insights for both academic and business sectors. We administered an online survey targeting consumers involved in the sharing economy across the U.S. and China. Identical surveys were distributed to participants in both countries, with localized language for the Chinese context. The survey comprised seven sections designed to assess respondents’ understanding of the sharing economy and their utilitarian, hedonic, and symbolic values, as well as their subjective norm, personal norm, attitude, and intention to participate.

The data for this study were gathered in September 2017 in the U.S. and in May 2020 in China, during the COVID-19 pandemic. The pandemic caused significant disruptions across industries, yet China emerged as the first major economy to recover, with most cities returning to normal by the second or third quarter of 2020 (Wang et al., 2022). An Overview of the Development of China’s Sharing Economy from 2017 to 2020 (State Information Center, 2021) reported year-on-year growth of approximately 2.9% in 2020, despite a deceleration compared to the previous year. Therefore, the data collection years reflect meaningful points in each country’s sharing economy development.

Independent datasets were collected using two online platforms: Amazon Mechanical Turk (MTurk) in the U.S., and Wenjuanxing, a professional online survey provider, in China. In both countries, participants spent approximately 7 min completing the survey.

After data collection, we conducted data analysis using SPSS 27.0 and AMOS 28. To evaluate the validity and reliability of the latent constructs, Confirmatory Factor Analysis (CFA) was performed on the final measurement model, which included 23 items. To test the proposed hypotheses, Structural Equation Modeling (SEM) was used. SEM is well-suited for multivariate path analysis and enables examination of complex relationships among variables beyond the capabilities of traditional regression analysis.

### 3.2 Measures

All measurement items used in this study were adapted from previously validated scales (see Table 1). Because the original items were developed in English, the Chinese version of the survey was translated using the back-translation method to ensure linguistic and conceptual equivalence.

**Table 1:** Measurement items and sources.

Variable	Item	Adapted from
Utilitarian value	Collaborative consumption saves me money.	Hwang and Griffiths (2017)
	Collaborative consumption is highly likely to get the proposed economic benefits.	
	Collaborative consumption would provide the proposed economic benefits what I have wanted.	
Hedonic value	I enjoy collaborative consumption.	Hwang and Griffiths (2017)
	Collaborative consumption makes me feel good.	
Symbolic value	Collaborative consumption makes me feel smart.	Hwang and Griffiths (2017)
	Collaborative consumption makes me feel more responsible.	
	Collaborative consumption makes me feel as a part of a larger cultural movement.	
Subjective norms	Most people who are important to me think that I should consume collaboratively.	Roos and Hahn (2017)
	The people in my life whose opinion I value would approve of consuming collaboratively.	
	Most people who are important to me consume collaboratively.	
	Many people like me consume collaboratively.	
Personal norm	I strongly feel a personal obligation to consume collaboratively.	Roos and Hahn (2017)
	I expect from myself to consume collaboratively.	
	Personally, I have a moral obligation to consume collaboratively.	
Attitude	All things considered; I find participating in collaborative consumption to be a wise move.	Roos and Hahn (2017)
	All things considered; I think collaborative consumption is a positive thing.	
	All things considered; I think participating in collaborative consumption is a good thing.	
Behavioral intention	All things considered; I expect to continue collaborative consumption often in the future.	Bhattacherjee (2001)
	I can see myself engaging in collaborative consumption more frequently in the future.	
	I can see myself increasing my collaborative consumption activities if possible.	
	It is likely that I will frequently participate in collaborative consumption communities in the future.	

The value dimensions (utilitarian, hedonic, and symbolic) are based on Hwang and Griffiths (2017). Utilitarian value was measured with a three-item scale assessing perceived economic benefit (e.g., “Collaborative consumption saves me money”). Hedonic value was measured with two items reflecting enjoyment and emotional satisfaction (e.g., “I enjoy collaborative consumption”). Symbolic value was assessed with three items addressing sustainability and identity (e.g., “Collaborative consumption makes me feel smart”).

Subjective norm and personal norm were measured using scales from Roos and Hahn (2017). Subjective norm was measured with four items reflecting social pressure and perceived expectations from significant others (e.g., “Most people who are important to me think that I should consume collaboratively”). Personal norm was measured with three items assessing internalized moral obligation (e.g., “I strongly feel a personal obligation to consume collaboratively”).

Attitude was measured using a three-item scale adapted from Roos and Hahn (2017), evaluating cognitive, emotional, and behavioral responses toward collaborative consumption (e.g., “All things considered, I find participating in collaborative consumption to be a wise move”).

Behavioral intention was measured using four items from Bhattacherjee (2001), capturing future engagement likelihood (e.g., “All things considered, I expect to continue collaborative consumption often in the future”).

All items used a 7-point Likert scale ranging from 1 (“strongly disagree”) to 7 (“strongly agree”). Table 1 summarizes the measurement items and their sources.

## 4 Results

### 4.1 Descriptive statistics and measurement model evaluation

Finally, a total of 302 responses were collected from U.S. participants, comprising 47.68% males (*n* = 144) and 52.32% females (*n* = 158). In China, 319 individuals responded; after removing questionnaires with extremely long completion times and uniform responses (indicative of disengagement), 305 valid responses remained. The gender distribution in the Chinese sample was also balanced, with females representing 52.46% (*n* = 160) and males 47.54% (*n* = 145). Table 2 present the demographic information of the samples.

**Table 2:** The general characteristics of the respondents.

Classification	Characteristic	Frequency	Percentage (%)
(a) U.S.			
Gender	Male	144	47.68
	Female	158	52.32
Age(years)	Under 18	16	5.30
	18–30	129	42.72
	31–40	86	28.48
	41 and over	71	23.50
Education	Senior high school or below	3	0.99
	University or college graduates	116	38.41
	Postgraduate or above	177	58.61
	None	6	1.99
(b) China			
Gender	Male	145	47.54
	Female	160	52.46
Age (years)	Under 18	5	1.64
	18–30	188	61.64
	31–40	91	29.84
	41 and over	21	6.89
Education	Senior high school or below	34	11.15
	University or college graduates	236	77.38
	Postgraduate or above	26	8.52
	None	9	2.95

Hair et al. (2018) suggest that a factor loading of at least 0.5 is a general guideline for practical significance (Hair et al., 2018). To evaluate convergent validity, all standardized factor loadings were checked and found to exceed the minimum threshold of 0.50. Internal consistency was assessed using Cronbach’s alpha, Composite Reliability (CR), and Average Variance Extracted (AVE). Additionally, the calculation of CR indicated that the CR values in both countries are within the acceptable range, meeting Hair et al.’s (2018) criterion that CR should be above 0.7. In the U.S. sample, CR values ranged from 0.867 to 0.952, and in the China sample from 0.800 to 0.869, all exceeding the 0.70 threshold. For convergent validity, the average variance extracted (AVE) should exceed 0.5 (Fornell and Larcker, 1981). In the study, AVE values ranged from 0.619 to 0.831 in the U.S., and from 0.506 to 0.690 in China, meeting the recommended 0.50 minimum. These results indicate that the measurement model demonstrates sufficient convergent validity in both samples.

Discriminant validity was assessed using Fornell and Larcker’s (1981) criteria. The square root of each construct’s AVE exceeded its correlations with other constructs, indicating acceptable discriminant validity and no concerns with multicollinearity in the U.S. sample (see Table 3). However, in the Chinese sample (Table 3), hedonic value, attitude, and intention showed some overlap, suggesting slightly weaker discriminant validity (Anderson and Gerbing, 1988).

**Table 3:** Means, standard deviations, and correlations among the key variables in the study.

Latent construct	Mean	SD	CR	AVE	1	2	3	4	5	6	7
(a) U.S.											
PER	4.205	1.531	0.900	0.750	**0.866** ^**a**^						
UTIL	5.413	1.140	0.899	0.749	0.592	**0.865** ^**a**^					
HEDO	5.215	1.331	0.899	0.816	0.654	0.705	**0.904** ^**a**^				
SYMB	4.932	1.338	0.870	0.692	0.786	0.792	0.811	**0.832** ^**a**^			
ATTI	5.642	1.166	0.952	0.831	0.531	0.778	0.852	0.738	**0.912** ^**a**^		
SUB	4.643	1.221	0.867	0.619	0.727	0.663	0.693	0.718	0.623	**0.787** ^**a**^	
INT	5.824	1.334	0.937	0.789	0.629	0.777	0.863	0.754	0.866	0.695	**0.888** ^**a**^
(b) China											
PER	4.863	1.124	0.869	0.689	**0.830** ^**a**^						
UTIL	5.000	0.925	0.800	0.584	0.324	**0.764** ^**a**^					
HEDO	5.370	0.952	0.711	0.552	0.675	0.323	**0.742** ^**a**^				
SYMB	4.668	1.088	0.787	0.553	0.684	0.432	0.680	**0.743** ^**a**^			
ATTI	5.635	0.854	0.786	0.552	0.523	0.299	0.748	0.404	**0.745** ^**a**^		
SUB	4.785	1.059	0.815	0.596	0.654	0.379	0.698	0.679	0.502	**0.772** ^**a**^	
INT	5.291	0.862	0.803	0.506	0.613	0.465	0.783	0.603	0.755	0.645	**0.711** ^**a**^

PER, Personal norm; UTIL, Utilitarian value; HEDO, Hedonic value; SYMB, Symbolic value; ATTI, Attitude; SUB, Subjective norms; INT, Behavioral intention.

a Square Root of AVE.

A commonly used guideline for the Comparative Fit Index (CFI) is values above 0.9 (Bentler, 1990). Model fit was evaluated using common indices: *χ*^2^/df < 3, GFI > 0.80, AGFI > 0.80, CFI > 0.90, RMSEA < 0.08. These indices suggested that the model showed a good fit to the data, with the normed chi-square was below the recommended 3.0 level (Hu and Bentler, 1999). The U. S. model achieved good fit: *χ*^2^ = 423.707, df = 152, χ^2^/df = 2.788, GFI = 0.882, AGFI = 0.821, CFI = 0.969, RMSEA = 0.077. The China model also showed good fit: *χ*^2^ = 385.521, df = 153, *χ*^2^/df = 2.520, GFI = 0.882, AGFI = 0.821, CFI = 0.912, RMSEA = 0.071.

### 4.2 Structural model evaluation and hypothesis testing

Maximum likelihood estimation was used to determine the parameters of the proposed structural relationship. Structural Equation Modeling (SEM) verified that the overall model fit was acceptable (see Table 4), enabling hypothesis testing based on the proposed path relationships. The structural models demonstrated a good fit, with the overall goodness-of-fit indices meeting the required thresholds.

**Table 4:** Fit indices: recommended versus actual values.

Fit indices	*χ*^2^/df	GFI	AGFI	CFI	RMSEA
Recommended value	<3	>0.80	>0.80	>0.90	<0.08
The U.S.	2.779	0.870	0.823	0.951	0.077
China	2.538	0.867	0.820	0.901	0.071

In the U.S. sample, results indicated that both utilitarian and hedonic values had significant positive effects on attitude toward the sharing economy. Specifically, utilitarian value (*β* = 0.415, *p* < 0.001) and hedonic value (*β* = 0.656, *p* < 0.001) were both significant, thereby supporting hypotheses H1a and H1b. However, symbolic value exhibited a non-significant negative effect on attitude (*β* = −0.110, *p* = 0.201), resulting in the rejection of H1c.

With regard to the influence of values on norms, utilitarian value did not significantly predict either subjective norm (*β* = 0.128, *p* = 0.174) or personal norm (*β* = −0.044, *p* = 0.203), leading to the rejection of H2a and H2b. Similarly, hedonic value did not have a significant effect on subjective norm (*β* = 0.181, *p* = 0.061) or personal norm (*β* = 0.071, *p* = 0.428), resulting in H3a and H3b also being unsupported. In contrast, symbolic value showed a significant positive impact on both subjective norm (*β* = 0.457, *p* < 0.001) and personal norm (*β* = 0.764, *p* < 0.001), providing support for hypotheses H4a and H4b.

The model also tested the effects of attitude and norms on behavioral intention. Attitude and subject norm significantly influenced behavioral intention (*β* = 0.739, *p* < 0.001), (*β* = 0.158, *p* = 0.009), supporting hypotheses H5 and H6. However, personal norm did not have a significant effect on behavioral intention (*β* = 0.060, *p* = 0.308), so H7 was not supported. These findings suggest that in the U.S. context, while utilitarian and hedonic values are critical in shaping attitudes, symbolic value plays more significant role in shaping both types of norms. Furthermore, attitude remains the most powerful predictor of behavioral intention.

In the Chinese sample, the results similarly supported hypotheses H1a and H1b, indicating that both utilitarian value (*β* = 0.237, *p* = 0.004) and hedonic value (*β* = 0.918, *p* < 0.001) had significant positive effects on attitude toward the sharing economy. However, contrary to expectations, symbolic value had a significant but negative effect on attitude (*β* = −0.263, *p* = 0.032), resulting in the rejection of H1c. Regarding the influence of utilitarian value on norms, the results did not show significant effects on either subjective norm (*β* = 0.092, *p* = 0.167) or personal norm (*β* = 0.059, *p* = 0.368), leading to the rejection of H2a and H2b. In contrast, hedonic value significantly influenced both subjective norm (*β* = 0.391, *p* < 0.001) and personal norm (*β* = 0.450, *p* < 0.001), thereby supporting H3a and H3b. Similarly, symbolic value was found to significantly predict both subjective norm (*β* = 0.475, *p* < 0.001) and personal norm (*β* = 0.552, *p* < 0.001), supporting H4a and H4b. When examining the predictors of behavioral intention, attitude (*β* = 0.551, *p* < 0.001) and personal norm (*β* = 0.327, *p* = 0.009) had significant effects, confirming H5 and H7, respectively. However, subjective norm did not significantly affect behavioral intention (*β* = 0.146, *p* = 0.174), resulting in the rejection of H6. These findings suggest that in the Chinese context, hedonic and symbolic values play a more prominent role in shaping both norms and attitudes, and that personal moral obligations may be more influential than external social expectations in driving behavioral intention (see Table 5).

**Table 5:** Summary of hypothesis testing.

Hypothesis	Statement	Hypothesis support	
		The U.S.	China
H1a	Utilitarian value positively affects attitude toward the benefits of the sharing economy.	Supported	Supported
H1b	Hedonic value positively affects attitude toward the benefits of the sharing economy.	Supported	Supported
H1c	Symbolic value positively affects attitude toward the benefits of the sharing economy.	Not supported	Not supported
H2a	Utilitarian value positively affects subjective norm toward the benefits of the sharing economy.	Not supported	Not supported
H2b	Utilitarian value positively affects personal norm toward the benefits of the sharing economy.	Not supported	Not supported
H3a	Hedonic value positively affects subjective norm toward the benefits of the sharing economy.	Not supported	Supported
H3b	Hedonic value positively affects personal norm toward the benefits of the sharing economy.	Not supported	Supported
H4a	Symbolic value positively affects subjective norm toward the benefits of the sharing economy.	Supported	Supported
H4b	Symbolic value positively affects personal norm toward the benefits of the sharing economy.	Supported	Supported
H5	Attitude positively affects behavioral intention toward the benefits of the sharing economy.	Supported	Supported
H6	Subjective norm positively affects behavioral intention toward the benefits of the sharing economy.	Supported	Not supported
H7	Personal norm positively affects behavioral intention toward the benefits of the sharing economy.	Not supported	Supported

## 5 Discussion

Grounded in the value–attitude–behavior (VAB) framework and the Theory of Reasoned Action (TRA), and the theory of planned behavior (TPB) this study examines how consumers’ utilitarian, hedonic, and symbolic values influence their attitudes, norms, and behavioral intentions toward participating in the sharing economy in the U.S. and China. Our findings confirm that attitudes and norms significantly shape consumers’ acceptance of the sharing economy. By comparing two cultural contexts, we uncover how these psychological mechanisms differ across countries. Overall, the results provide both theoretical and practical insights, which are discussed below.

### 5.1 Theoretical implications

This study contributes to the literature on the sharing economy by emphasizing the role of individual-level values and norms in shaping participation. It underscores the importance of integrating values into models of consumer behavior to better understand sharing intentions. It also provides new evidence for applying the TRA and the TPB across different cultural contexts.

First, we provide a comparative assessment of how different value dimensions influence attitudes in both countries. The results show that utilitarian and hedonic values significantly shape consumer attitudes in the U.S. and China, which aligns with the core proposition of the VAB framework that values drive attitude (Homer and Kahle, 1988; Milfont et al., 2010). This reinforces the notion that the efficient use of resources is a universally appealing value. For example, shared mobility services in China attract users by reducing transaction costs (Shao et al., 2023). Similarly, previous research in collectivist cultures has shown that functional benefits like cost-efficiency and resource optimization predict green purchase intentions (Cheung and To, 2019). In the U.S., utilitarian motivations such as affordability and convenience also influence attitudes, paralleling mechanisms found in other domains (Fu et al., 2014).

Additionally, consumers in both countries report deriving enjoyment from their participation, confirming the role of hedonic value. This finding is consistent with previous studies that also emphasize the hedonic motives behind participation on sharing platforms (Kozlenkova et al., 2021; von Richthofen, 2022). However, symbolic value did not influence attitudes in either country. That is an outcome consistent with Hwang and Griffiths’s (2017) findings. This may reflect the utilitarian nature of sharing platforms, where users are motivated more by functional or emotional benefits than by identity signaling. For instance, Bardhi and Eckhardt (2012) found that Zipcar users value on-demand access and cost savings over symbolic associations, and that such motivations may affect attitude indirectly through sustainability perceptions (Lange et al., 2023). In the context of peer-to-peer accommodation, symbolic value negatively influenced continuance intention (Ofori et al., 2023). These empirical observations collectively support the basic idea that a person’s real behavior is influenced by both values and attitudes (Tajeddini et al., 2021).

Second, the influence of cultural context becomes especially apparent when examining how values affect norms. In both countries, utilitarian value did not significantly affect either subjective or personal norm. Symbolic value, by contrast, shaped both types of norms in both contexts. Notably, hedonic value influenced neither type of norm in the U.S., but had a strong positive effect on both subjective and personal norms in China. These results illustrate how cultural divergence moderates the value–norm link, which aligns with prior research, where values influence behavior via more specific and tangible constructs like subjective norms (Izquierdo-Yusta et al., 2022).

Third, the findings point to key differences in how norms influence behavioral intention. In the U.S., subjective norm significantly predicted intention, whereas personal norm did not. This finding aligns with prior research showing that social influence and trend affinity shape Airbnb users’ intentions in Western contexts (So et al., 2018). Conversely, in China, subjective norm had no significant effect on intention, while personal norm did. This may be because Chinese consumers rely more on information-based social influence, such as online reviews from strangers, than on normative influence from friends or family (Wang and Hassan, 2024). These dynamics reduce the predictive power of subjective norm and elevate the role of internalized moral obligation in shaping behavioral intention.

### 5.2 Practical implications

This study also offers practical guidance for sharing economy platforms seeking to adapt to different cultural environments. Understanding the role of values and norms can help firms tailor their marketing and platform design strategies.

In the U.S. market, enhancing subjective norm is crucial. Platforms should leverage social proof mechanisms, such as reviews, influencer endorsements, and peer referrals, to amplify social influence. And emphasize social media as a potent platform for spreading normative messages via user-generated content (Liu and Lapinski, 2025). They should also create emotionally engaging experiences that align hedonic value with symbolic identity, for instance, by promoting narratives like “sharing is freedom” or “smart and sustainable choices.”

In the Chinese market, activating personal norms through responsibility-based messaging is more effective. Firms should emphasize narratives around environmental protection, community benefit, or national development. Moreover, platforms should enhance the social aspects of consumption. For example, firms could incorporate features that support group participation or sharing stories to strengthen the relationship between hedonic and symbolic value and personal norms (Song et al., 2025).

### 5.3 Limitations and future research

While this study contributes novel insights into cross-cultural sharing behavior, it also presents several limitations that should be addressed in future research.

First, the analysis focuses exclusively on the U.S. and China. Expanding the model to include consumers in other regions (e.g., Europe or Southeast Asia) would improve generalizability and enable broader comparisons of cultural influences. Additionally, future studies could explore sharing behaviors across diverse sectors, including consumer goods, media, and communications (Lim, 2020). Additionally, we intend to use a mixed-methods approach, combining online surveys with offline interviews and field studies, to reduce sampling bias and improve data representativeness. Lastly, utilizing longitudinal tracking data will help confirm the causal links between values, attitudes, norms, and behavioral intentions.

Second, although participants were real consumers in the sharing economy, we did not examine how demographic characteristics affect the relationships in the model. Prior studies suggest that social norms may influence behavior differently depending on the consumer segment (Sands et al., 2020). Future research could examine how factors such as age, gender, or lifestyle shape the relationships among values, norms, and behavioral intention.

Finally, our model focused specifically on the role of values, attitudes, and norms. While these are important personal-level predictors, other well-documented factors (e.g., trust, risk perception, or reputation) were outside the scope of this study. Incorporating these additional constructs in future studies could provide a more comprehensive understanding of what drives sharing behavior. Moreover, using qualitative methods such as interviews or ethnographic observation may offer richer insights into the psychological and contextual drivers of sharing economy participation.

## 6 Conclusion

In this study, we examined how the three value dimensions influence consumers’ attitudes, norms, and behavioral intentions toward sharing economy participation through cross-cultural comparisons between the U.S. and China, using the value–attitude–behavior (VAB) framework, and the theory of reasoned action (TRA) and the theory of planned behavior (TPB). We found that utilitarian and hedonic values are the main determinants of consumers’ attitudes in both countries, while symbolic value has no significant effect on attitudes in either context. Additionally, symbolic value consistently influences both subjective and personal norms in both settings. Conversely, hedonic value strongly positively affects both types of norms in China but has no significant impact in the U.S. Furthermore, the influence of norm on behavioral intention shows distinct cultural differences: subjective norm acts as the primary predictor in the U.S., whereas personal norm is the main driver of behavioral intention among Chinese consumers. Future research will explore other cultural contexts to test the cross-cultural applicability of the model. We also plan to employ a mixed-methods approach, combining online surveys with offline interviews and field studies, to reduce sampling bias and enhance data representativeness. Finally, using longitudinal tracking data will help verify the causal relationships between values, attitudes, norms, and behavioral intentions.

## Data Availability

The data supporting the conclusions of this study are included in the article. Further inquiries can be directed to the corresponding author.
